# Supplementary material for: Gut microbiota Turicibacter strains differentially modify bile acids and host lipids
Source: Nat Commun. 2023 Jun 20;14:3669. doi: 10.1038/s41467-023-39403-7 (PMC10281990; doi:10.1038/s41467-023-39403-7)
Supplement: Supplementary file 1 — Supplementary Information [file 41467_2023_39403_MOESM1_ESM.pdf]

SUPPLEMENTARY FIGURES

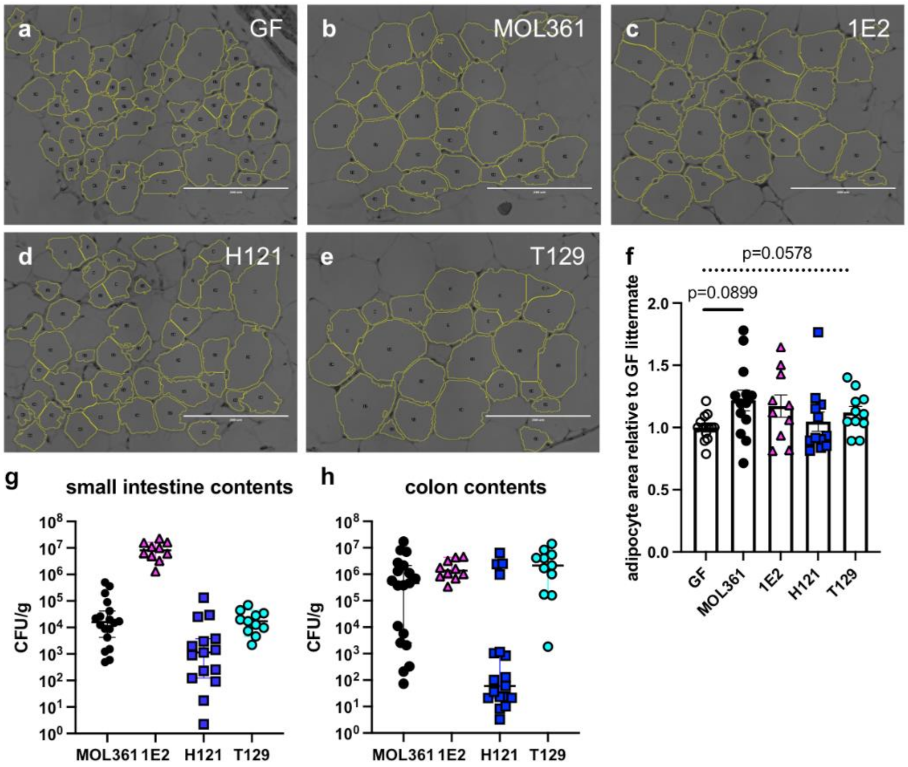

Supplementary Fig. 1. Strain-dependent variation in colonization and adipocyte size. a-e, Representative images of adipose tissue from *Turicibacter* monocolonized mice. **f**, Sex- and litter- matched relative adipocyte area of mice monocolonized with individual *Turicibacter* strains. Each point represents mean of 10 images of adipocyte area per animal. **g, h**, Colony-forming units (CFU) equivalents per gram of contents for distal small intestine (**g**) or proximal colon **(h).** Each dot represents sample from one animal, per colonization n for qPCR: MOL361=18, 1E2=10, H121=15, T129=11; for adipocyte are calculation, n: GF=14, MOL361=13, 1E2=10, H121=12, T129=11. for adipocyte area calculation. Statistics for adipocyte area performed by Welch's ANOVA with Dunnet's multiple comparisons, dotted bar represents ANOVA statistic for each group versus the combined experimental mean. Error bars are mean +/-SEM. Data are provided as source data file.

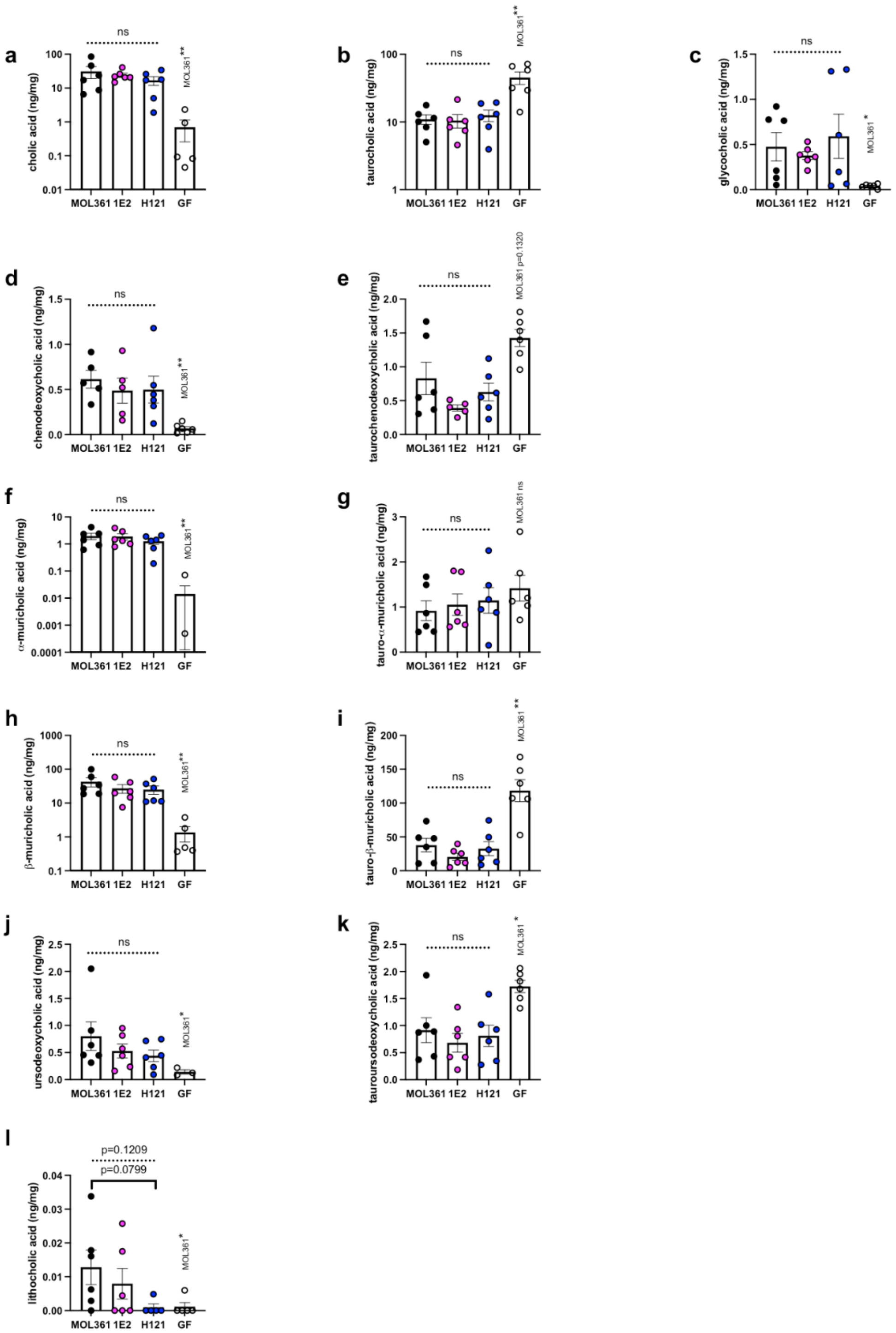

Supplementary Fig. 2. *Turicibacter* colonization alters cecal bile acids. Cecal concentrations of conjugated and unconjugated species of **a-c,** cholic acid, **d-e**, chenodeoxycholic acid, **f-g**, alpha-muricholic acid, **h-i**, beta-muricholic acid, **j-k**, ursodeoxycholic acid, and the unconjugated secondary bile acid **l**, lithocholic acid, in GF or *Turicibacter* monocolonized mice. Each point represents one animal, n=6 animals per condition. Statistical comparisons between *Turicibacter* isolates performed with Kruskal-Wallis test with Dunn's multiple comparisons, GF-MOL361 comparisons with Mann-Whitney test. Dotted line represents combined intra-*Turicibacter* ANOVA p-value, value written above GF represents GF-MOL361. Error bars are mean +/-SEM p-value, *p<0.05, **p<0.005. p-values for each comparison (inter-*Turicibacter* ANOVA/H121-MOL361/H121-1E2/GF-MOL361): S2a=0.5378/0.7734/0.5585/0.0087;

S2b=0.6203/0.999/0.6338/0.0043; S2c=0.999/0.999/0.999/0.0087; S2d=0.4527/0.3887/0.8346/0.0022; S2e=0.6955/0.999/0.999/0.1320; S2f=0.6226/0.7159/0.7739/0.0043; S2g=0.6522/0.7159/0.999/0.1797; S2h=0.4838/0.4684/0.999/0.0087; S2i=0.4929/0.999/0.6608/0.0043; S2j=0.4479/0.3883/0.999/0.0238; S2k=0.7761/0.999/0.999/0.0260; S2l=0.1209/0.0799/0.5637/0.0455. Data are provided as source data file.

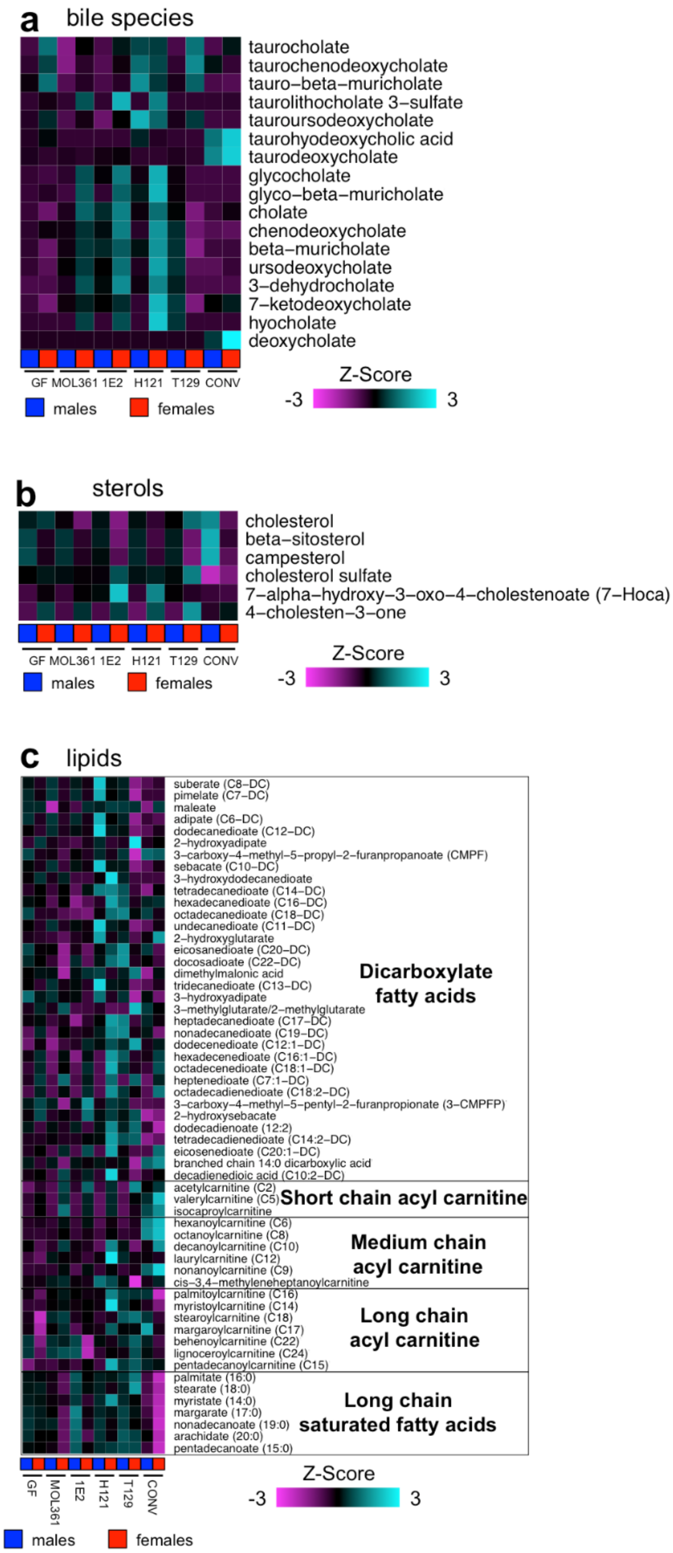

Supplementary Fig. S3. Host effects of *Turicibacter* colonization varies with sex. a-c, Heatmap of mean relative **a**) bile species, **b**) sterols, or **c**) lipids. Column labels represent colonization state and sex, blue=male, red=female. n=3-6 per group, except female T129 (n=1).

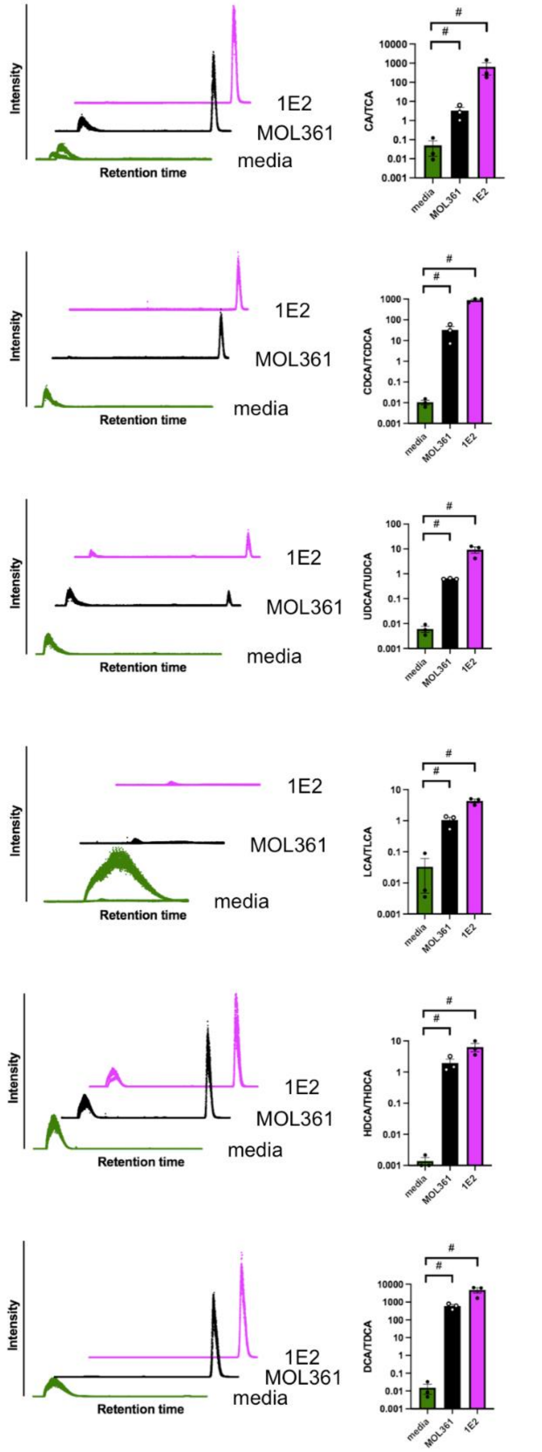

Supplementary Fig. 4. ***Turicibacter*** **strains MOL361 and 1E2 can deconjugate at least six taurine-conjugated bile acids.** Chromatograms (left) and unconjugated/conjugated bile acid ratios (right) of *Turicibacter* MOL361 or 1E2 grown for 24 hours in YCFA + 0.5mM of individual taurine-conjugated bile acids: **a**, TCA; **b**, TCDCA; **c**, tauroursodeoxycholic acid (TUDCA); **d**, taurolithocholic acid (TLCA); **e**, taurohyodeoxycholic acid (THDCA); **f**, taurodeoxycholic acid (TDCA). Chromatograms are concatenated reconstructed chromatograms for conjugated and unconjugated bile acid. Each trace and each point represents one biological replicate, n=3 cultures. Statistics were performed by Mann-Whitney test, #p=0.1 for all comparisons shown (Note: this p-value is the minimum for this test and our experimental

parameters). Data are provided as source data file.

Isolate/ 7α-HSDH	Seq. ID %	Seq. Coverage
MOL361	57%	100%
18F6	57%	100%
T46	57%	100%
GALT-E2	57%	100%
H121	59%	98%
T129	59%	98%
GALT-G1	59%	98%
1E2	32%	95%
TA25	32%	95%

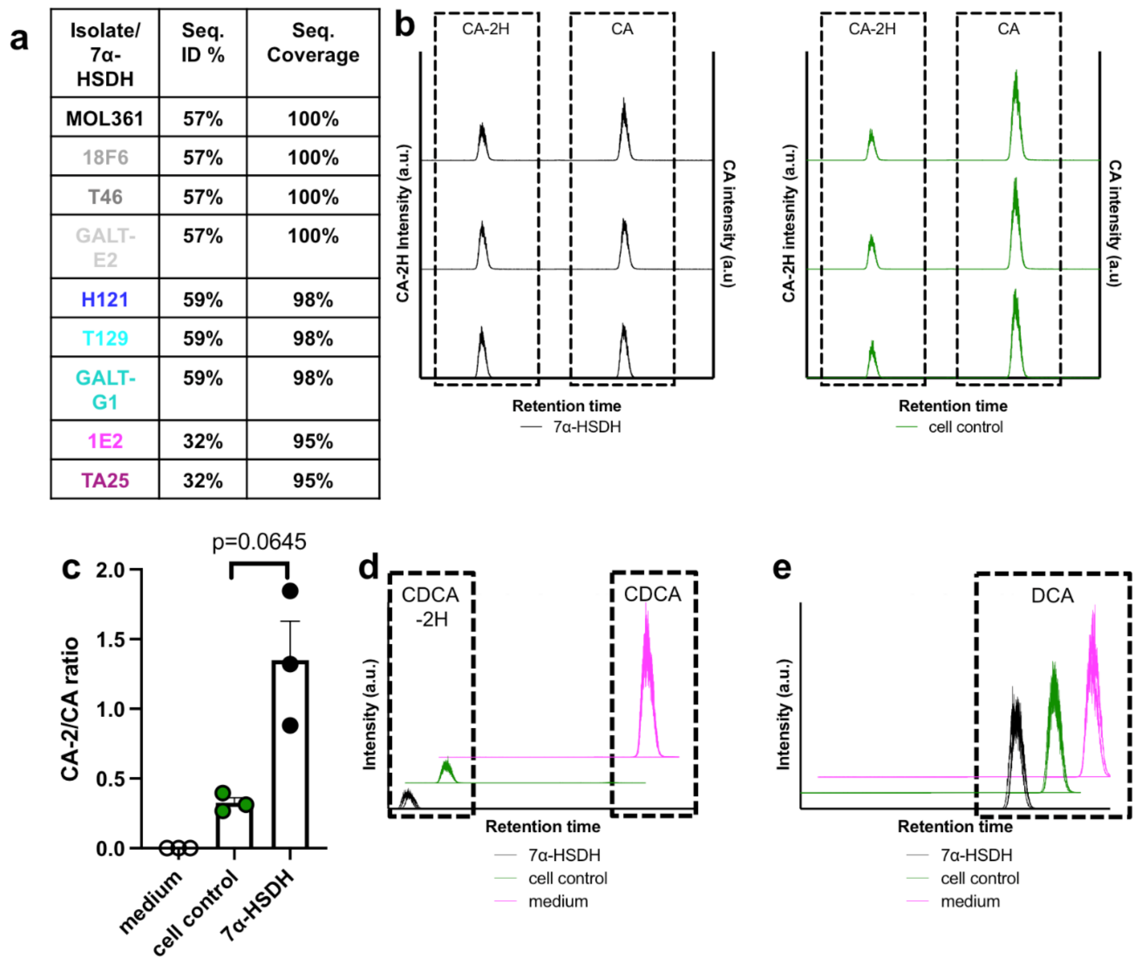

Supplementary Fig. 5. Some strains of *Turicibacter* encode a functional 7alpha-HSDH gene. **a**, Table depicting amino acid similarity and sequence coverage between closest predicted 7alpha-HSDH homolog in noted isolate genome and 7alpha-HSDH gene from *Clostridium absonum*. **b**, Chromatograms of cholic acid (CA) or cholic acid with 2 hydrogens removed (CA-2H) from *E. coli* cultures expressing the MOL361 HSDH homolog (7alpha-HSDH) or non-bile modifying gene sequence in the same plasmid (cell control). Chromatograms from independent triplicate cultures are shown, dotted boxes indicate each bile species. **c**, Quantification of CA/CA-2H ratios determined from reconstructed areas under the curve in **b**. Statistical comparison performed with Welch's t-test, n=3 cultures, bars indicate mean +/- SEM. **d**, Same as **b**, but with CDCA instead of CA. **e**, same as **b**, but with DCA instead of CA. **d** and **e** triplicate chromatograms are stacked on the same axis. Data are provided as source data file.

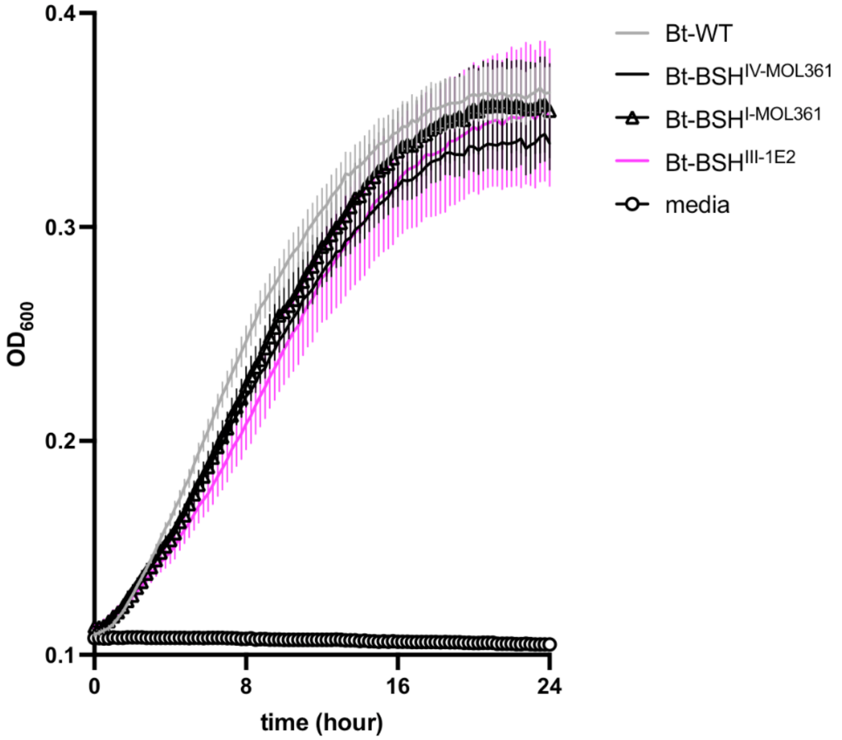

Supplementary Fig. 6. Expressing *bsh* genes from *Turicibacter* does not impart significant *in vitro* growth defect of *B. thetaiotaomicron*. OD_{600} readings of noted *B. thetaiotaomicron* strains in BHI-S medium. Each point represents mean +/- SEM for 6 independent cultures. Data are provided as source data file.

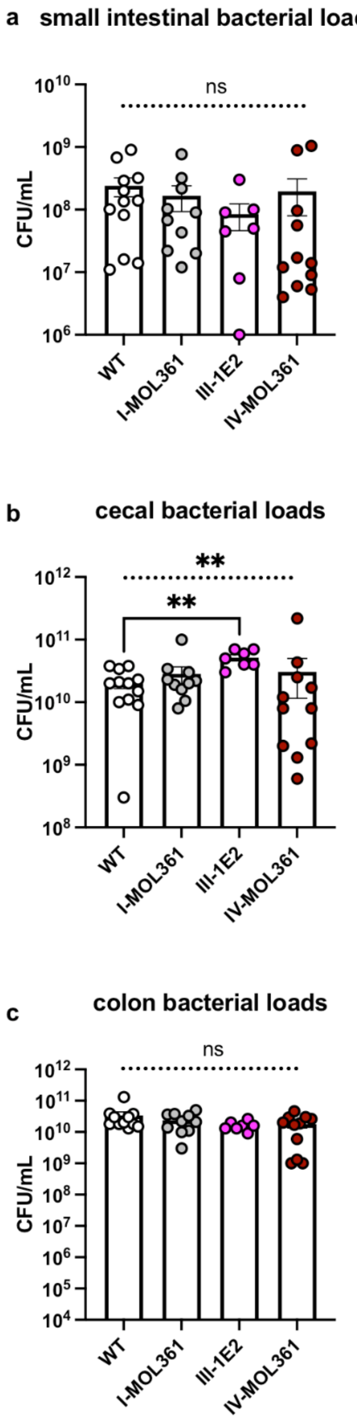

Supplementary Fig. 7. *bsh* expression does not alter *B. thetaiotaomicron* colonization of the murine gut. CFU/mL quantifications of the noted *B. thetaiotaomicron* strains in the **a,** distal small intestine; **b,** cecum and; **c,** proximal colon of gnotobiotic mice. n for eachgroup: *Bt*-WT=12, *Bt*-BSH-MOL361-I=10, *Bt*-BSH-1E2-III=7, *Bt*-BSH-MOL361-IV=11. Comparisons with Welch's ANOVA test and Dunnet's multiple comparisons. Error bars are mean +/-SEM**.** p-values (ANOVA/WT vs. BSHI-MOL361/WT vs. BSHIII-1E2/WT vs. BSHIV-MOL361): S9a=0.6647/0.8725/0.2642/0.9834; S9b=0.0041/0.7395/0.0029/0.9248; S9c=0.2578/0.7621/0.2983/0.4202. Data are provided as source data file.

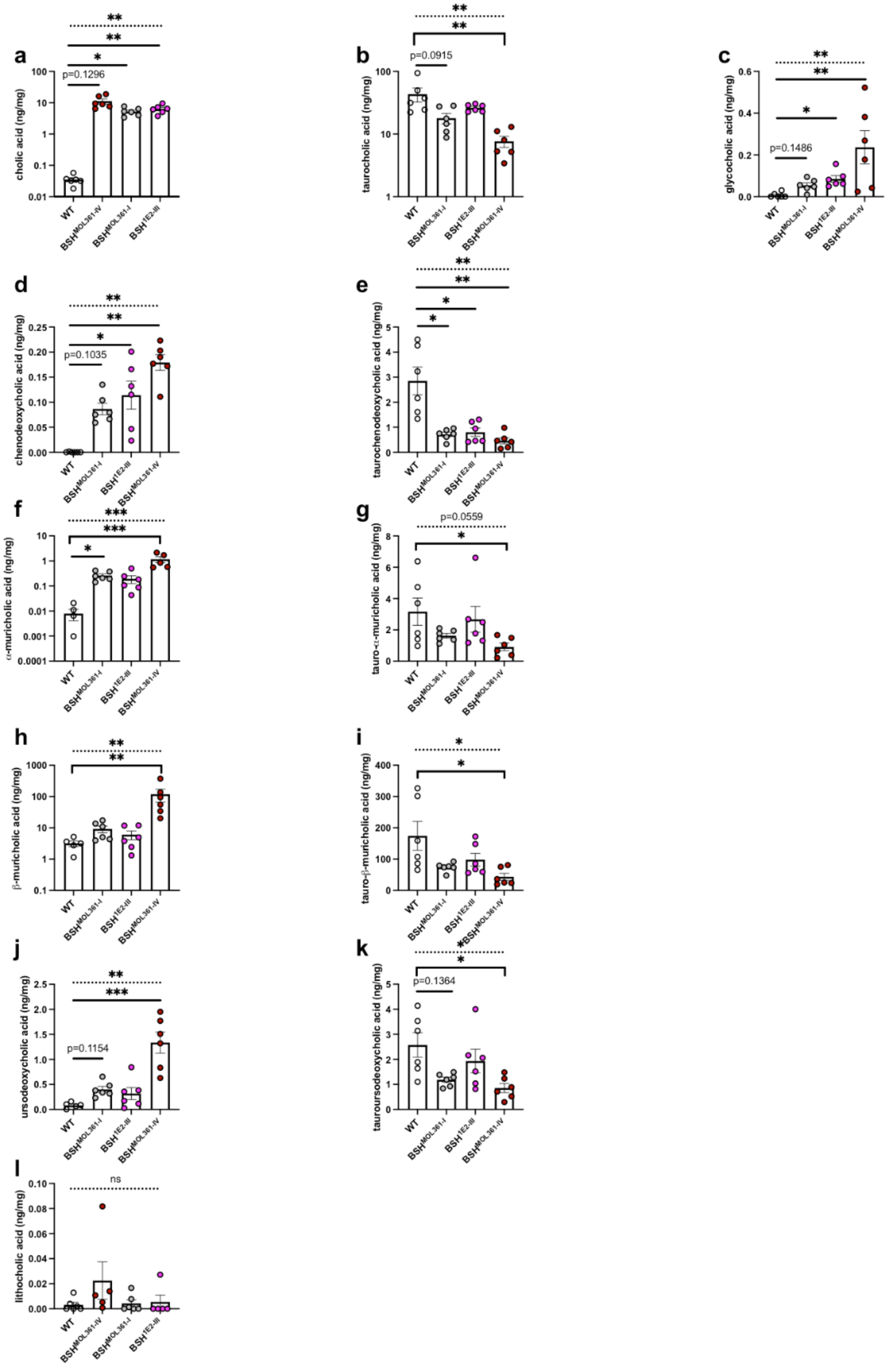

Supplementary Fig. 8. Engineered *B. thetaiotaomicron* transforms host bile acids *in vivo*. Cecal abundance of unconjugated and conjugated species of primary bile acids **a-c,** cholic acid, **d-e**, chenodeoxycholic acid, **f-g**, alpha-muricholic acid, **h-i**, beta-muricholic acid, **j-k**, ursodeoxycholic acid, and the unconjugated secondary bile acid **l**, lithocholic acid. n=3 (3 males, 3 females) for each condition. Statistics performed with Kruskal-Wallis test with Dunn's multiple comparisons. Dotted horizontal line represents total ANOVA statistic across all four groups. Error bars are mean +/-SEM *p<0.05, **p<0.005, ***p<0.0005. p-values for each comparison (combined ANOVA/WT vs. BSHI-MOL361/WT vs. BSHIII-1E2/WT vs. BSHIV-MOL361): S10a=0.0006/0.1296/0.0211/0.0001; S10b=0.0012/0.0915/0.999/0.0006; S10c=0.0032/0.1486/0.0085/0.0021; S10d=0.0006/0.1095/0.0231/0.0001;

S10e=0.0026/0.0239/0.0303/0.0010; S10f=0.0003/0.0480/0.3074/0.0001; S10g=0.0559/0.999/0.999/0.0429; S10h=0.0030/0.8110/0.999/0.0028; S10i=0.0232/0.2825/0.5743/0.0066; S10j=0.0037/0.3927/0.9816/0.0011; S10k=0.0151/0.1364/0.999/0.0076; S10l=0.1543/0.999/0.999/0.1464. Data are provided as source data file.

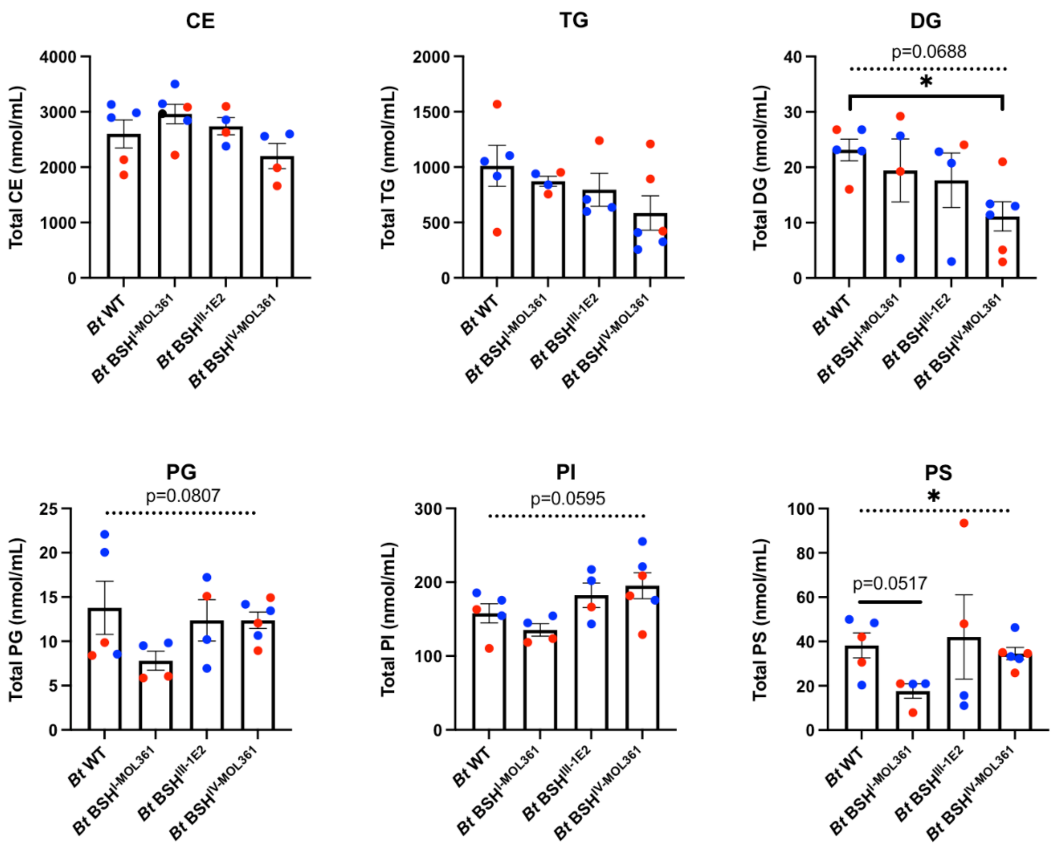

Supplementary Fig. 9. *Turicibacter bsh* expression by *B. thetaiotaomicron* is sufficient to drive broad scale changes in circulating host lipids in male and female mice. Related to **Fig. 6e-j**, circulating absolute concentrations of specific lipid categories in mice colonized by *B. thetaiotaomicron* expressing the noted *Turicibacter bsh* homologs. Each point represents one animal, red points indicate female, blue represent male. n per colonization: *Bt*-WT=5 (3M, 2F), *Bt*-BSH-MOL361-I=4 (2M, 2F), *Bt*-BSH-1E2-III=4 (3M, 1F), *Bt*-BSH-MOL361-IV=6 (3M, 3F) Error bars represent mean +/- SEM. Statistical comparison done with Welch's ANOVA with Dunnett's multiple comparisons, dotted bar represents ANOVA statistic for all four groups. Error bars are mean +/-SEM *p<0.05. p-values for each lipid group (combined ANOVA/WT vs. BSHI-MOL361/WT vs. BSHIII-1E2/WT vs. BSHIV-MOL361): CE=0.1850/0.5935/0.9494/0.5922; TG=0.3970/0.8469/0.7495/0.2872; DG=0.0688/0.8966/0.6860/0.0152; 0.0807/0.2818/0.9735/0.9541; PI=0.0595/0.4343/0.6081/0.3049; PS=0.0295/0.0517/0.9960/0.9068. Data are provided as source data file.

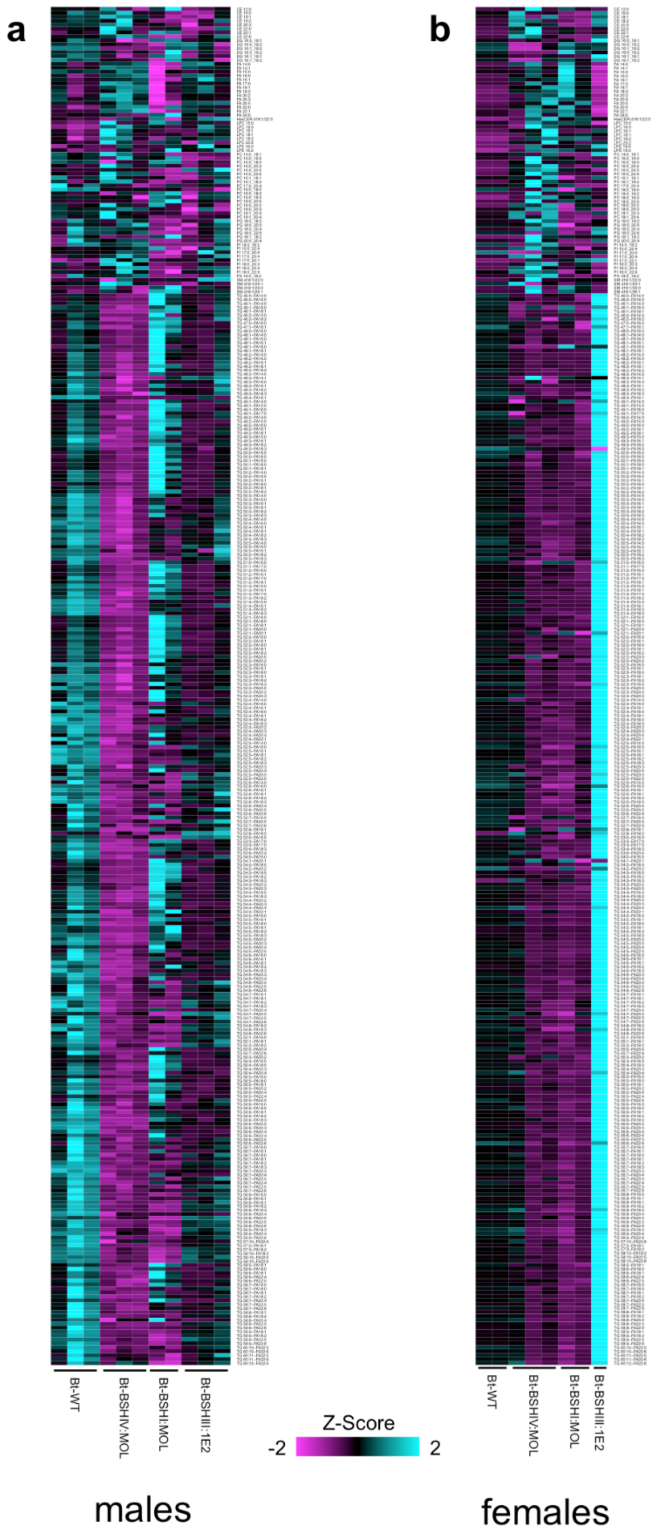

Supplementary Fig. 10. Sex differences in lipidomic response to *Turicibacter bsh* expression in *B. thetaiotaomicron*. Similar to **Fig. 5b**, but animal lipidome analysis separated into **a**) males and **b**) females. Note that lipid species presented were found to be significantly altered by expression of at least one *bsh* in males and females combined (i.e. all lipid species shown across the three analyses are the same). Each column represents one animal.

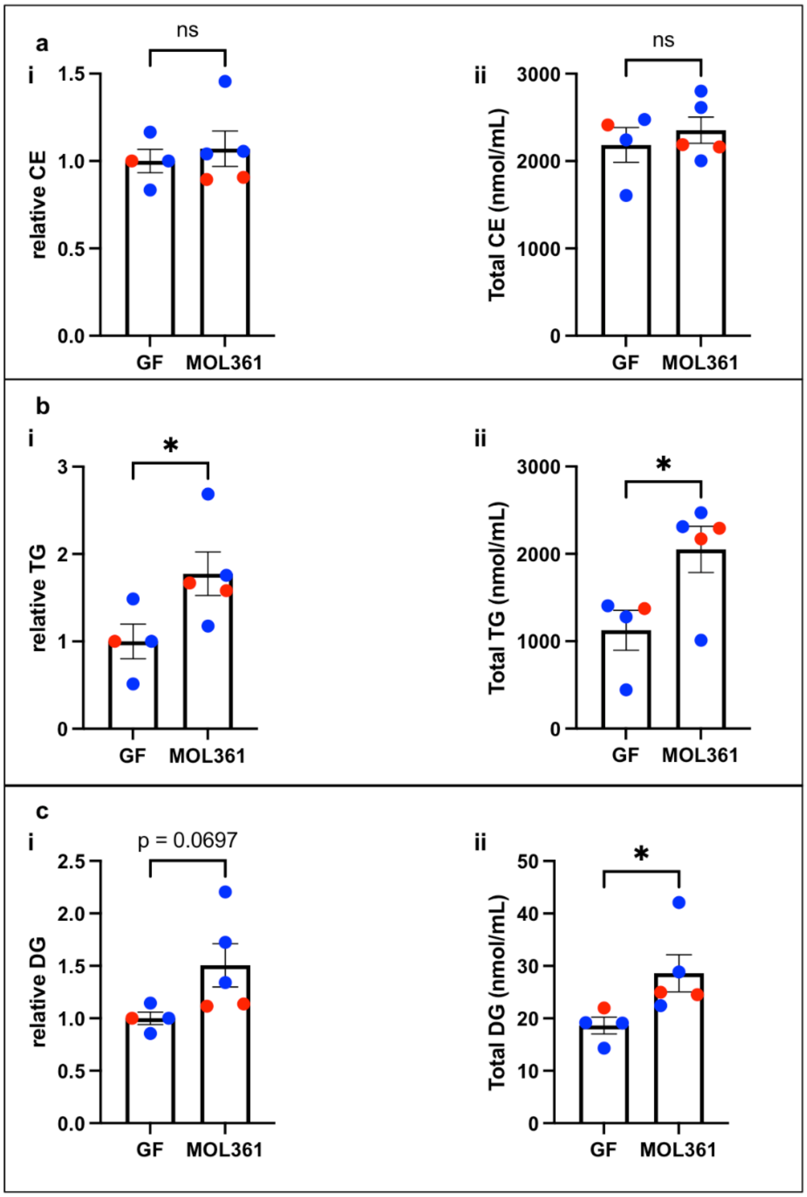

Supplementary Fig. 11. *T. sanguinis* MOL361 colonization leads to increased triglycerides and diacylglycerides, consistent with adipose tissue increases. Plasma levels of combined **a**, cholesterol esters, **b,** triglycerides, and **c,** diacylglycerides. **i:** relative to sex-matched GF littermates, **ii**: absolute quantification. n per condition: GF=4 (3M, 1F), MOL361=5 (3M, 2F). red=female, blue=male. Statistical comparisons Student's two tailed t-test with Welch's correction. Error bars are mean +/-SEM *p<0.05. p-values: ai=0.5815; aii=0.5245; bi=0.454; bii=0.0330; ci=0.0697; cii=0.0463. Data are provided as source data file.

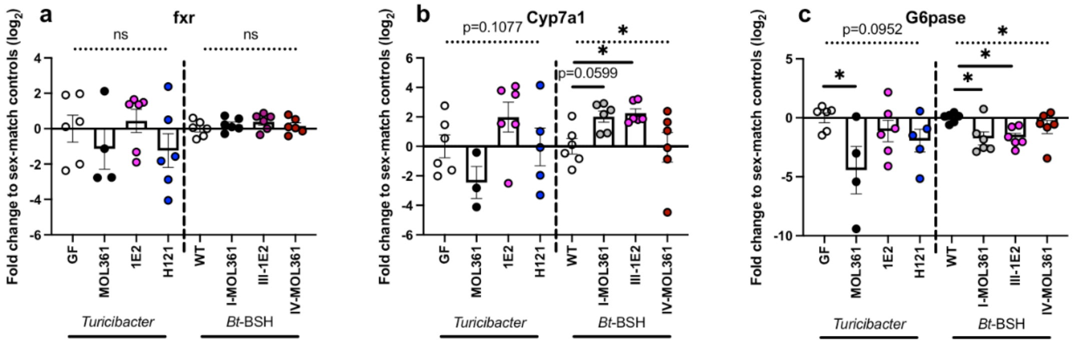

Supplementary Fig. 12. *Turicibacter* colonization and *bsh* expression induce similar gene expression patterns in the liver. qRT-PCR analysis of liver transcript levels of **a,** Fxr; **b,** Cyp7a1; and **c,** G6pase after colonization with the noted *Turicibacter* strains or *B. thetaiotaomicron* expressing the noted *bsh* gene. Data are displayed as fold change relative to appropriate control (GF for *Turicibacter* colonizations, *Bt*-WT for *bsh* colonizations). Each point represent data from a single animal, n=6 for all conditions except fxr-MOL36 (n=4), Cyp7a1-MOL361 (n=3), Cyp7a1-H121 (n=5), G6pase-MOL361 (n=4), G6pase-H121 (n=5). Statistics performed with Kruskal-Wallis test with Dunn's multiple comparisons. Dotted horizontal line represents ANOVA statistic for that comparison of groups below the line. Error bars are mean +/-SEM, *p<0.05. p-values for each comparison (GF-*Turicibacter* ANOVA/GF-MOL361/GF-1E2/GF-H121/*Bacteroides* BSH ANOVA/*Bt*-WT vs. *Bt*-BSHI-MOL361/*Bt*-WT vs. *Bt*-BSHIII-1E2/*Bt*-WT vs. *Bt*-BSHIV-MOL361):
fxr=0.5986/0.999/0.999/0.999/0.4899/0.9816/0.3927/0.999;
Cyp7a1=0.1077/0.5266/0.5630/0.999/0.0193/0.0599/0.0383/0.999;
G6pase=0.0952/0.0422/0.8538/0.3811/0.0352/0.0480/0.0341/0.8110. Data are provided as source data file.
