# Supplementary material for: Gut microbiota Turicibacter strains differentially modify bile acids and host lipids
Source: Nat Commun. 2023 Jun 20;14:3669. doi: 10.1038/s41467-023-39403-7 (PMC10281990; doi:10.1038/s41467-023-39403-7)
Supplement: Supplementary file 3 — Supplementary Data Descriptions [file 41467_2023_39403_MOESM3_ESM.docx]

**File name: Supplementary Data 1**

**Description: Table of bile transformations performed by Turicibacter isolates.** Table indicating presence/absence of noted bile species after growth in mixture of bile acids described in Fig. 4b.

**File name: Supplementary Data 2**

**Description: Bile transformations performed by B. thetaiotaomicron expressing Turicibacter bsh genes.** Table indicating presence/absence of noted bile species after growth in mixture of bile acids described in Fig. 6a, b.

**File name: Supplementary Data 3**

**Description: Description of animals used in this work.**

**File name: Supplementary Data 4**

**Description: List of strains used in this work.**

**File name: Supplementary Data 5**

**Description: Serum metabolomics from GF, CONV, and *Turicibacter* monocolonized mice.** Volume-adjusted log-transformed levels of listed serum metabolites from mice from different colonization states. Raw p-values generated from Mann-Whitney U test (GF-MOL361 and MOL361-CONV) or Kruskal-Wallis (MOL361-1E2-H121) test. Adjusted p-values represent p-values with Šidák correction for number of tests of that metabolite.

**File name: Supplementary Data 6**

**Description: Raw and normalized e/g WAT values for *Turicibacter* and *B. thetaiotaomicron*-colonized mice.**

**File name: Supplementary Data 7**

**Description: Absolute quantification of lipid species from plasma of mice colonized with *B. thetaiotaomicron* engineered to express *Turicibacter bsh* genes, GF mice, or MOL361 monocolonized mice.** Raw p-values generated from Student’s t-test comparison with Bt-WT for *Bacteroides* conditions or comparison between GF and MOL361. Adjusted p-values represent p-values with Šidák correction for number of tests of that lipid.

**File name: Supplementary Data 8**

**Description: List of oligos used in the work.**
