# Supplementary material for: Gut microbiota Turicibacter strains differentially modify bile acids and host lipids
Source: Nat Commun. 2023 Jun 20;14:3669. doi: 10.1038/s41467-023-39403-7 (PMC10281990; doi:10.1038/s41467-023-39403-7)
Supplement: Supplementary file 5 — Reporting Summary [file 41467_2023_39403_MOESM5_ESM.pdf]

Corresponding author(s): Jonathan B. Lynch
Last updated by author(s): 5/31/2023

Reporting Summary

Nature Portfolio wishes to improve the reproducibility of the work that we publish. This form provides structure for consistency and transparency in reporting. For further information on Nature Portfolio policies, see our Editorial Policies and the Editorial Policy Checklist.

Statistics

For all statistical analyses, confirm that the following items are present in the figure legend, table legend, main text, or Methods section.

n/a	Confirmed	
☐	☒	The exact sample size (*n*) for each experimental group/condition, given as a discrete number and unit of measurement
☐	☒	A statement on whether measurements were taken from distinct samples or whether the same sample was measured repeatedly
☐	☒	The statistical test(s) used AND whether they are one- or two-sided<br>*Only common tests should be described solely by name; describe more complex techniques in the Methods section.*
☐	☒	A description of all covariates tested
☐	☒	A description of any assumptions or corrections, such as tests of normality and adjustment for multiple comparisons
☐	☒	A full description of the statistical parameters including central tendency (e.g. means) or other basic estimates (e.g. regression coefficient) AND variation (e.g. standard deviation) or associated estimates of uncertainty (e.g. confidence intervals)
☐	☒	For null hypothesis testing, the test statistic (e.g. *F*, *t*, *r*) with confidence intervals, effect sizes, degrees of freedom and *P* value noted<br>*Give P values as exact values whenever suitable.*
☒	☐	For Bayesian analysis, information on the choice of priors and Markov chain Monte Carlo settings
☒	☐	For hierarchical and complex designs, identification of the appropriate level for tests and full reporting of outcomes
☒	☐	Estimates of effect sizes (e.g. Cohen's *d*, Pearson's *r*), indicating how they were calculated

Our web collection on statistics for biologists contains articles on many of the points above.

Software and code

Policy information about availability of computer code

Data collection	No software was used for data collection.
Data analysis	anvi'o (v.7.1) was used for genomic sequence comparisons. OrthoANIu (https://www.ezbiocloud.net/tools/ani) was used to calculate avergae nucleotide identity. R (v.3.6.3) package pheatmap was used to generate heatmaps. CLC Genomics Workbench v(20.0.3) (Qiagen) was used to assemble genomes and perform gene/amino acid sequence comparisons. Fiji (ImageJ v.2.1.0) plugin Adiposoft (v1.1.16) was used to calculate adipocyte area. Manufacturer-provided software was used to measure mass spectrometry peak area. Unless otherwise noted, Prism (v9.3.1) or Excel (v14.7.1) was used for statistical analysis. tblastn (v 20.12.0) was used for 7alpha HSDH sequence comparisons.

For manuscripts utilizing custom algorithms or software that are central to the research but not yet described in published literature, software must be made available to editors and reviewers. We strongly encourage code deposition in a community repository (e.g. GitHub). See the Nature Portfolio guidelines for submitting code & software for further information.

Data

Policy information about availability of data

All manuscripts must include a data availability statement. This statement should provide the following information, where applicable:

- Accession codes, unique identifiers, or web links for publicly available datasets
- A description of any restrictions on data availability
- For clinical datasets or third party data, please ensure that the statement adheres to our policy

Metabolomics and lipidomics datasets are attached to this manuscript as supplementary tables. Raw sequencing reads and genome assemblies for Turicibacter strains are deposited at NCBI under BioProject ID PRJNA846348. Metabolomics datasets are deposited at Metabolights as project MTBLS7921 (https://www.ebi.ac.uk/metabolights/editor/study/MTBLS7921).

Human research participants

Policy information about studies involving human research participants and Sex and Gender in Research.

Reporting on sex and gender	N/A
Population characteristics	N/A
Recruitment	N/A
Ethics oversight	N/A

Note that full information on the approval of the study protocol must also be provided in the manuscript.

Field-specific reporting

Please select the one below that is the best fit for your research. If you are not sure, read the appropriate sections before making your selection.

☒ Life sciences ☐ Behavioural & social sciences ☐ Ecological, evolutionary & environmental sciences

For a reference copy of the document with all sections, see nature.com/documents/nr-reporting-summary-flat.pdf

Life sciences study design

All studies must disclose on these points even when the disclosure is negative.

Sample size	No statistical predictions were used to determine sample sizes. Sample sizes were based on comparison to previous study (Fung et al, 2019).
Data exclusions	When appropriate, outliers were removed from analysis using ROUT method with 1% cutoff, as recommended by our statistical analysis program. This only affected small number of cecal bile measurements. Original raw data are included in source files.
Replication	All animal experiments are pooled combinations of at least two independent experiments (i.e. different litters and cohorts, experiments performed on different days), except for CONV mice (males and females experimentally separate, but single litter and cohort). Mass spectrometry experiments were representative of at least three independent experiments performed on different days. Each data point represents independent biological replicates (i.e. individual mouse or individual bacterial culture).
Randomization	Animals were separated by litter and sex, notched by a blinded researcher, then randomly placed into same-sex conditions (i.e. litters could be combined, but sexes were kept separated). Images for adipocyte measurements were semi-randomly numbered (i.e. sequentially numbered with all samples collected that day, with continuous numbering between experiments), images were framed manually with only consideration being image quality, and adipocytes were measured automatically by program. Image quality control was performed by two blinded researchers.
Blinding	Animal samples were given semi-random number identifiers (see above) at the point of sample collection and maintained with those identifiers for the entirety of sample processing and data analysis. Researchers were blind to condition during data collection and statistical analysis was automated. Image analysis was performed through automated processing and blinded image quality control.

Reporting for specific materials, systems and methods

We require information from authors about some types of materials, experimental systems and methods used in many studies. Here, indicate whether each material, system or method listed is relevant to your study. If you are not sure if a list item applies to your research, read the appropriate section before selecting a response.

Materials & experimental systems

n/a	Involved in the study
☒	☐ Antibodies
☒	☐ Eukaryotic cell lines
☒	☐ Palaeontology and archaeology
☐	☒ Animals and other organisms
☒	☐ Clinical data
☒	☐ Dual use research of concern

Methods

n/a	Involved in the study
☒	☐ ChIP-seq
☒	☐ Flow cytometry
☒	☐ MRI-based neuroimaging

Animals and other research organisms

Policy information about studies involving animals; ARRIVE guidelines recommended for reporting animal research, and Sex and Gender in Research

Laboratory animals	6-8 week old (at start of experiment) male and female Swiss Webster mice, maintained at 22-25C with humidity control.
Wild animals	Did not involve wild animals
Reporting on sex	Male and female mice were used, and data are presented as combined values and sex-separated values where differences were noted. Number of each sex is noted where relevant.
Field-collected samples	Did not involved field samples.
Ethics oversight	All animal experiments were performed according to UCLA Institutional Animal Care and Use Committee-approved protocols.

Note that full information on the approval of the study protocol must also be provided in the manuscript.
